# Re-Evaluating Post-TAVR Antithrombotic Strategies in Nonanticoagulated Patients

**DOI:** 10.1016/j.jacadv.2025.101983

**Published:** 2025-07-07

**Authors:** Ioannis Skalidis, Thomas Hovasse, Francesca Sanguinetti, Philippe Garot, Mariama Akodad

**Affiliations:** Institut Cardiovasculaire Paris-Sud, Hôpital Jacques Cartier, Ramsay-Santé, Massy, France

The recent network meta-analysis by Siami et al^1^ offers timely insight into post-transcatheter aortic valve replacement (TAVR) antithrombotic therapy for patients without a baseline indication for anticoagulation.^1^ Their findings suggest that single antiplatelet therapy is associated with reduced bleeding without an increase in mortality or ischemic events compared to dual antiplatelet therapy or oral anticoagulants. This reinforces current guideline shifts toward simplified regimens.

Several aspects of the analysis, however, merit deeper exploration. First, while bleeding outcomes favored single antiplatelet therapy across subgroups, ischemic endpoints were largely comparable. Might this suggest that the residual thrombotic risk in this population is overestimated? Or that the low incidence of thromboembolism makes net clinical benefit largely driven by bleeding?

Second, the increased mortality observed with low-dose rivaroxaban plus antiplatelet therapy raises important mechanistic questions. Should we interpret this as class-specific harm or as a failure of dose modulation in this unique population?

Third, the identification of chronic obstructive pulmonary disease as a modifier of bleeding risk invites the possibility of comorbidity-based stratification. Could bleeding risk calculators incorporating noncardiac variables refine post-TAVR treatment selection more effectively than valve type alone?

Fourth, while the analysis pooled studies across varying valve types, procedural approaches, and follow-up durations, it remains unclear whether valve-specific factors—such as leaflet thrombosis rates or radial force differences between balloon-expandable and self-expanding platforms—might influence antithrombotic risk–benefit balance. Could a stratified analysis by valve technology help identify subgroups where more or less intensive regimens are warranted?

Siami et al^1^ have set a foundation for rethinking antithrombotic strategy post-TAVR. We believe further clarification on patient phenotyping and procedural context may help optimize therapy while minimizing harm.
